# Clinical performance and utility of a comprehensive next-generation sequencing DNA panel for the simultaneous analysis of variants, TMB and MSI for myeloid neoplasms

**DOI:** 10.1371/journal.pone.0240976

**Published:** 2020-10-19

**Authors:** Nikhil Shri Sahajpal, Ashis K. Mondal, Sudha Ananth, Allan Njau, Pankaj Ahluwalia, Kimya Jones, Meenakshi Ahluwalia, Nwogbo Okechukwu, Natasha M. Savage, Vamsi Kota, Amyn M. Rojiani, Ravindra Kolhe

**Affiliations:** 1 Department of Pathology, Medical College of Georgia, Augusta University, Augusta, GA, United States of America; 2 Department of Pathology, Aga Khan University Hospital, Nairobi, Kenya; 3 Department of Medicine, Medical College of Georgia, Augusta University, Augusta, GA, United States of America; The Jackson Laboratory for Genomic Medicine, UNITED STATES

## Abstract

The extensively employed limited-gene coverage NGS panels lead to clinically inadequate molecular profiling of myeloid neoplasms. The aim of the present investigation was to assess performance and clinical utility of a comprehensive DNA panel for myeloid neoplasms. Sixty-one previously well characterized samples were sequenced using TSO500 library preparation kit on NextSeq550 platform. Variants with a VAF ≥ 5% and a total read depth of >50X were filtered for analysis. The following results were recorded-for clinical samples: clinical sensitivity (97%), specificity (100%), precision (100%) and accuracy (99%) whereas reference control results were 100% for analytical sensitivity, specificity, precision and accuracy, with high intra- and inter-run reproducibility. The panel identified 880 variants across 292 genes, of which, 749 variants were in genes not covered in the 54 gene panel. The investigation revealed 14 variants in ten genes, and at least one was present in 96.2% patient samples that were pathogenic/ likely pathogenic in myeloid neoplasms. Also, 15 variants in five genes were found to be pathogenic/ likely pathogenic in other tumor types. Further, the TMB and MSI scores ranged from 0–7 and 0–9, respectively. The high analytical performance and clinical utility of this comprehensive NGS panel makes it practical and clinically relevant for adoption in clinical laboratories for routine molecular profiling of myeloid neoplasms.

## Introduction

Myeloid malignancies are characterized by uncontrolled proliferation and/or defects in differentiation of abnormal myeloid progenitor cells. Myelodysplastic syndromes (MDS) and myeloproliferative neoplasms (MPNs) are often thought to be precursors to a higher grade myeloid malignancies, namely acute myeloid leukemia (AML) [1]. AML is characterized by clonal expansion of myeloid precursors (i.e. blasts), resulting in impaired hematopoiesis and bone marrow failure [2]. The pathogenesis of AML has been well studied using cytogenetic analysis for more than three decades [3, 4]. However, nearly 50% of AML patients have normal karyotype at diagnosis, and many of these genomes lack structural abnormalities even when assessed with fluorescent in-situ hybridization (FISH) or single nucleotide polymorphism (SNP) arrays [5–7]. The cytogenetic heterogeneity of AML remains well recognized, however, the enormous molecular heterogeneity is only beginning to become evident over the past few years. The National Comprehensive Cancer Network (NCCN) guidelines for AML diagnosis recommends cytogenetic analysis using karyotype and FISH, in addition to molecular analysis for at least *c-KIT*, *FLT3* (ITD and TKD), *NPM1*, *CEBPA* (biallelic), *IDH1*, and *IDH2* genes. The field of genomics in myeloid malignancies, and related implications in AML are evolving rapidly [8]. Current guidelines recommend these patients be tested using multiplex gene panels and next-generation sequencing (NGS) analysis for comprehensive prognostic assessment.

In this context, clinical laboratories have incorporated NGS platform(s) for routine clinical screening of myeloid neoplasm samples. However, many laboratories have used relatively small targeted panels that screen prominent mutation hotspots in ≤ 50 genes. Although this approach is cost- and time- effective with minimal data analysis and reporting complexity, it yields an incomplete mutational profile, omitting several important known hotspot mutations [9, 10]. Molecular profiling by NGS methodology has already introduced a paradigm shift in detecting pathogenic variants, however, recognizing the extensive molecular heterogeneity of these neoplasms, there is a dire need to investigate these tumors on a high-throughput NGS platform for comprehensive genetic screening. The molecular heterogeneity of AML was highlighted in a recent whole exome sequencing (WES) based study by The Cancer Genome Atlas (TCGA) Research Network on 200 AML patients, which identified complex interplay of genetic alterations with 13 mutations detected per patient across 237 genes [11].

In addition to the sequencing variants [single nucleotide variants (SNVs), insertion deletions (Indels)], parameters such as tumor mutational burden (TMB) and microsatellite instability (MSI) are providing additional screening parameters that may correlate with responsiveness to checkpoint inhibitor immunotherapy [12]. Although TMB has previously been assessed by WES, recent studies suggest that TMB can also be calculated with targeted panels covering genomic content of ≥1.1 Mb and/or >300 genes. Additionally, targeted panels with larger genomic content (at least 1.5 Mb) perform well with samples containing less than 30 mutations/Mb [12, 13].

Thus, considering the need for comprehensive genetic testing that can be readily adopted in clinical laboratories, we sought to evaluate the clinical performance and utility of a comprehensive 523 gene NGS panel (Illumina, San Diego, US) for screening myeloid neoplasms. The high-throughput comprehensive NGS panel was validated for SNVs and indels/duplications in myeloid neoplasms. The assessment of SNVs, indels, TMB and MSI in a single assay, using only 120 ng DNA, provides comprehensive genomic analyses that is efficient in sample requirements and time- and cost-effective. The clinical utility of this comprehensive panel was also assessed by investigating/identifying novel variants in myeloid neoplasms. Further, the novel variants, TMB and MSI were correlated with clinical parameters.

## Materials and methods

### Samples

Sixty-one (61) well characterized samples were sequenced and evaluated in the present study. Of these, reference control samples viz. Seraseq Myeloid Mutation DNA (SeraCare Life Sciences, Milford, MA), AcroMetrix Oncology Hotspot Control (Thermo Scientific, Fremont, CA), and myeloid neoplasm controls were run at different concentrations for analytical performance calculations. Under the IRB approved protocol, forty (40) patient samples with confirmed myeloid neoplasms were included in this study. Of the forty patient samples included in this study, clinical information was available on 27 patients. The study was approved by the IRB A- BIOMEDICAL I (IRB REGISTRATION #00000150), Augusta University. HAC IRB # 611298. No consent was needed because it was a retrospective study. Based on the IRB approval, all PHI was removed and all data was anonymized before accessing for the study. The patients' medical records at Augusta University Medical Center, Augusta, GA were accessed during 01/20–02–20.

### DNA isolation

DNA was isolated from bone marrow aspirates using the QIAamp DNA Blood Mini kit (QIAGEN, Hilden, Germany) as per manufacturer’s protocol. Nanodrop spectrophotometer was used to analyze the DNA quality with an OD 260/280 value between 1.7 and 2.2 being considered acceptable. Double stranded DNA was measured using Qubit dsDNA broad range assay kit (#Q32850, Invitrogen, USA) and 120 ng gDNA was used for library preparation.

### Library preparation and sequencing

All samples passing quality control (QC) were subjected to library preparation using the hybrid capture-based TSO 500 library preparation kit (# 20028214, TruSight Oncology 500 DNA Kit, Illumina, San Diego, CA) following manufacturer’s instruction. In brief, the DNA was fragmented using an ultrasonicator (Covaris, Woburn, MA) with a target peak of ~130 bp. After end repair, A-tailing, and adapter ligation, the adapter ligated fragments were amplified using index PCR (UP-index) specific primers. Further, the libraries were enriched through hybrid capture based method using specific probes. This was followed by PCR based enrichment, cleanup, and quantification of double stranded DNA using high sensitivity Qubit (#Q32854 Invitrogen, USA) measurement. The libraries were subjected to bead based normalization and were sequenced using V2 sequencing reagent kits on a NextSeq550 platform (Illumina, San Diego, CA) as per manufacturer recommendations.

### Sequencing data analysis

The raw sequence reads were converted to FASTQ format using BaseSpace TSO 500 Assessment App (Illumina). The VCF files were analyzed using Basespace variant interpreter for SNVs and indels/duplications. Variants with a variant allele frequency (VAF) ≥ 5% and a total read depth of >50X were filtered for analysis. The variants were compared for concordance with previously reported variants, as the same samples were sequenced on a 54 gene myeloid panel, and reported through PierianDx reporting solution. The TMB and MSI were recorded from Basespace TSO 500 Assessment App. In brief, the 523-gene panel contains 1.94 Mb genomic content, though the performance of TMB detection is set by analyzing SNVs and indels in the coding regions, with sophisticated variant calling and germline filtering algorithms for enhanced accuracy. TMB = number of eligible somatic mutations per Mb (targeted region defined as high confidence regions with ≥ 50× coverage). For MSI, the algorithm analyzed 130 MSI marker sites to calculate a quantitative score.

### Performance metric evaluation

The performance metric was calculated for both clinical and reference control samples for SNVs and indels/duplications. Seven performance criteria viz. positive percentage agreement (PPA), negative percentage agreement (NPA), positive predictive value (PPV), negative predictive value (NPV), accuracy, false negative rate (FNR), and false positive rate (FPR) were evaluated.

### Limit of detection and reproducibility studies

The limit of detection (LOD) was assessed by sequencing the reference controls viz. Seraseq Myeloid Mutation DNA and AcroMetrix Oncology Hotspot Control by sequentially diluting with wild type DNA to provide different levels of dilutions (100% (triplicate), 50%, 25%, and 12.5%) and (100%, 62.5% (triplicate), 50%, 25% (triplicate), 12.5%, 10%, and 1%), respectively. The Seraseq Myeloid Mutation DNA 100% dilution and AcroMetrix Oncology Hotspot Control 25% dilution were sequenced in triplicate to evaluate intra-run reproducibility. Similarly, one clinical sample and AcroMetrix Oncology Hotspot Control at 62.5% dilution were sequenced in three different runs to evaluate inter-run reproducibility.

### Novel variants and clinical correlation

The variants with a VAF ≥ 5% and a total read depth of > 50X were filtered for analysis. In addition to the performance analysis, the data was analyzed for novel variants by performing a search in various knowledge bases such as COSMIC and ClinVar for pathogenic significance in myeloid neoplasms and/or other tumor types. Also, the novel pathogenic variants identified were correlated with clinical parameters. Of the forty patient samples included in this study, clinical information was available on 27 patients. The demographic and clinical parameters of patients included in this analyses are listed in Table 1. The clinicopathologic parameters included were: age, gender, ethnicity, survival, cytogenetic profile, and management.

**Table 1:** Clinical characteristics of patients included in the study.

Parameters/Characteristics	Groups	Number (n)
Patients		27
Age	<68	14
	>68	13
Sex	Male	17
	Female	10
Ethnicity	Caucasian	18
	African American	9
Classification	Acute myeloid leukemia (AML)	
	AML with mutated NPM1	5
	AML, NOS	7
	APL with PML-RARA	1
	Mixed phenotype acute leukemia (MPAL), T/myeloid, NOS	1
	AML with myelodysplasia-related changes	2
	Myeloid sarcoma	1
	Acute myeloid leukemia with monocytic differentiation	2
	Myelodysplastic syndromes (MDS)	
	MDS with excess blasts	2
	MDS with multilineage dysplasia	1
	MDS with ring sideroblasts (MDS-RS)	1
	Myeloproliferative neoplasms (MPN)	
	Primary myelofibrosis (PMF)	2
	Essential thrombocythemia (ET)	1
	Polycythemia vera (PV)	1
Survival	Deceased	4
	Alive	23
Cytogenetics*	Normal	13
	Abnormal	13
Management*	Transplant	7
	No transplant	19

*Information not available for one patient.

## Results

### Sequencing performance

**Fig 1:**
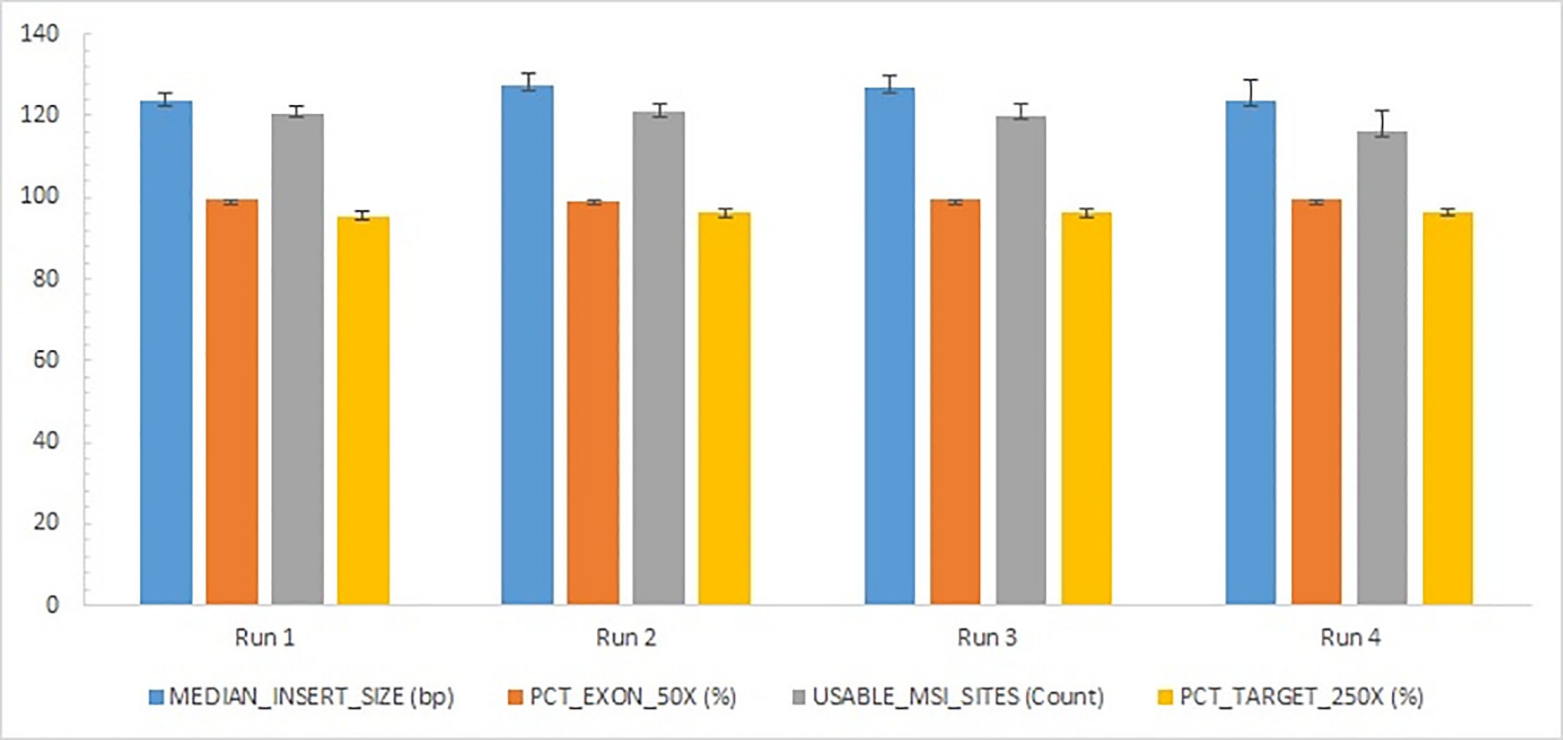
The run metrics for DNA library QC parameters. PCT: Percentage reads passing filter, MSI: Microsatellite Instability.

A typical sequencing run of TSO 500 performed on NextSeq550 platform consists of 10 samples. In this study, a total of 61 samples were sequenced in 9 runs. Forty (40) samples were sequenced in 4 runs containing only myeloid neoplasm samples, whereas the remaining samples were sequenced in 5 runs multiplexed with solid tumor samples. The average percentage reads passing filter (PCT_PF) for four representative runs was observed to be 90.1%. The percent base calls with a quality score of Q30 or higher for read 1 and 2 were 93.6 and 91.3, respectively. Four critical DNA library QC parameters were recorded as follows: the median insert size from the sequencing reads for the 4 runs was observed to be 125.3 bp; the percent of exon bases with coverage ≥ 50X and percent target bases with coverage >250X were observed to be 99.35 and 96.01, respectively; the average usable MSI counts were observed to be 119.3 (Fig 1). The run metrics parameters for DNA library QC for -contamination, -small variant calling and TMB, -MSI, and DNA expanded metrics met the recommended threshold values prescribed by the manufacturer (S1 Table).

### Performance metric evaluation/ analytical performance

The performance metric was calculated for both clinical and reference control samples for SNVs and indels/duplications. In clinical samples, the performance metric was calculated for a total of 78 variants (64 unique variants), which included 61 SNVs, and 17 indels/duplications. For the SNVs, the platform had a clinical sensitivity of 96.5%, detecting 59 of the 61 SNVs, specificity of 100%, precision of 100%, and accuracy of 99.8%. The two SNVs that were not reported were found in the read maps below the VAF cut-off of 5% [*CBL* p.C358Y (VAF = 3.45), *KRAS* p.G13D (VAF = 2.1)]. For indels/duplications, the platform had a clinical sensitivity, specificity, precision and accuracy of 100% (Table 2). However, it must be noted that Flt3 ITDs were not included for in evaluation/calculation.

**Table 2:** Performance metric evaluation in clinical samples.

Performance Criterion	Single nucleotide variants (SNVs)	*Indels/Duplication (Without Flt3 ITD)
PPA/ Sensitivity (%)	96.5	100
NPA/ Specificity (%)	100	100
PPV/ Precision (%)	100	100
NPV (%)	99.8	100
Accuracy (%)	99.8	100
FNR (%)	3.4	0
FPR (%)	0	0

Positive percentage agreement (PPA) = TP/ (TP+FN).

Negative percentage agreement (NPA) = TN/ (TN+FP).

Positive predictive value (PPV) = TP/ (TP+FP).

Negative predictive value (NPV) = TN/ (TN+FN).

Accuracy = TP+TN/All Results.

False Negative Rate (FNR) = FN/ (FN+TP).

False Positive Rate (FPR) = FP/ (FP+TN).

In reference control samples, the Seraseq myeloid mutation DNA consisted of 23 variants in 16 genes. Of these 23 variants, 13 were SNVs, and 10 were indels/duplicaions/ITD. The Seraseq Myeloid Mutation DNA was diluted (100%, 50%, 25%, and 12.5%) and performance metric was calculated for each dilution. The analytical sensitivity, specificity, precision and accuracy for SNV and indels/duplications detection using both Seraseq myeloid mutation DNA and AcroMetrix Oncology Hotspot Control, was found to be 100%. These performance metrics were also found to decrease with dilution of the sample (Table 3). The list of variants detected by the platform used for calculating performance metric are listed in S2 Table.

**Table 3:** Performance metric evaluation in Seraseq myeloid mutation DNA, AcroMetrix oncology hotspot control and myeloid neoplasm control samples.

Variant Type	Dilution	PPA %	NPA%	PPV %	NPV %	Accuracy %	FNR %	FPR %
Seraseq myeloid mutation DNA (SNVs)	100%	100	100	100	100	100	0	0
	100%	100	100	100	100	100	0	0
	100%	100	100	100	100	100	0	0
	50%	42.8	100	100	84	85.7	57.1	0
	25%	0	100	0	75	75	100	0
	10%	0	100	0	75	75	100	0
Seraseq myeloid mutation DNA *Indels/duplications (Without Flt3 ITDs, and CALR)	100%	100	100	100	100	100	0	0
	100%	85.7	100	100	75	90	14.2	0
	100%	85.7	100	100	75	90	14.2	0
	50%	14.2	100	100	33.3	40	85.7	0
	25%	0	100	100	30	30	100	0
	10%	0	100	100	100	100	0	0
AcroMetrix Oncology Hotspot Control (SNVs)	100%	100	100	100	100	100	0	0
	62.5%	100	100	100	100	100	0	0
	62.5%	85.7	100	100	98	98.2	14.2	0
	62.5%	85.7	100	100	98	98.2	14.2	0
	50%	14.2	100	100	89	89.2	85.7	0
	25%	0	100	100	87.5	87.5	100	0
	25%	0	100	100	87.5	87.5	100	0
	25%	0	100	100	87.5	87.5	100	0
	12.5%	0	100	100	87.5	87.5	100	0
	10%	0	100	100	87.5	87.5	100	0
	1%	0	100	100	87.5	87.5	100	0
Myeloid neoplasm control samples (SNVs)	100%	100	100	100	100	100	0	0

Positive percentage agreement (PPA) = TP/ (TP+FN).

Negative percentage agreement (NPA) = TN/ (TN+FP).

Positive predictive value (PPV) = TP/ (TP+FP).

Negative predictive value (NPV) = TN/ (TN+FN).

Accuracy = TP+TN/All Results.

False Negative Rate (FNR) = FN/ (FN+TP).

False Positive Rate (FPR) = FP/ (FP+TN).

### Limit of detection and reproducibility studies

**Fig 2:**
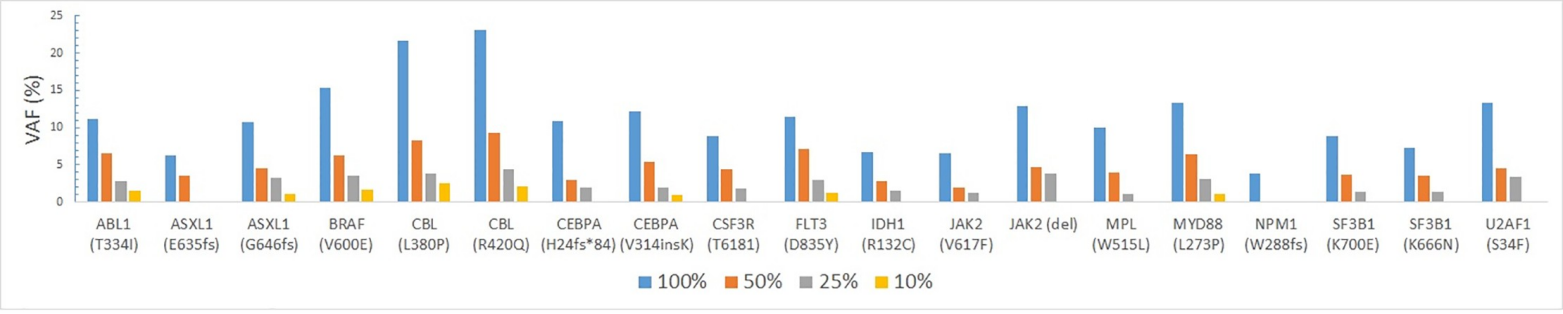
Limit of detection (LOD) study using Seraseq Myeloid Mutation DNA sequentially diluted to 50%, 25% and 10%.

**Fig 3:**
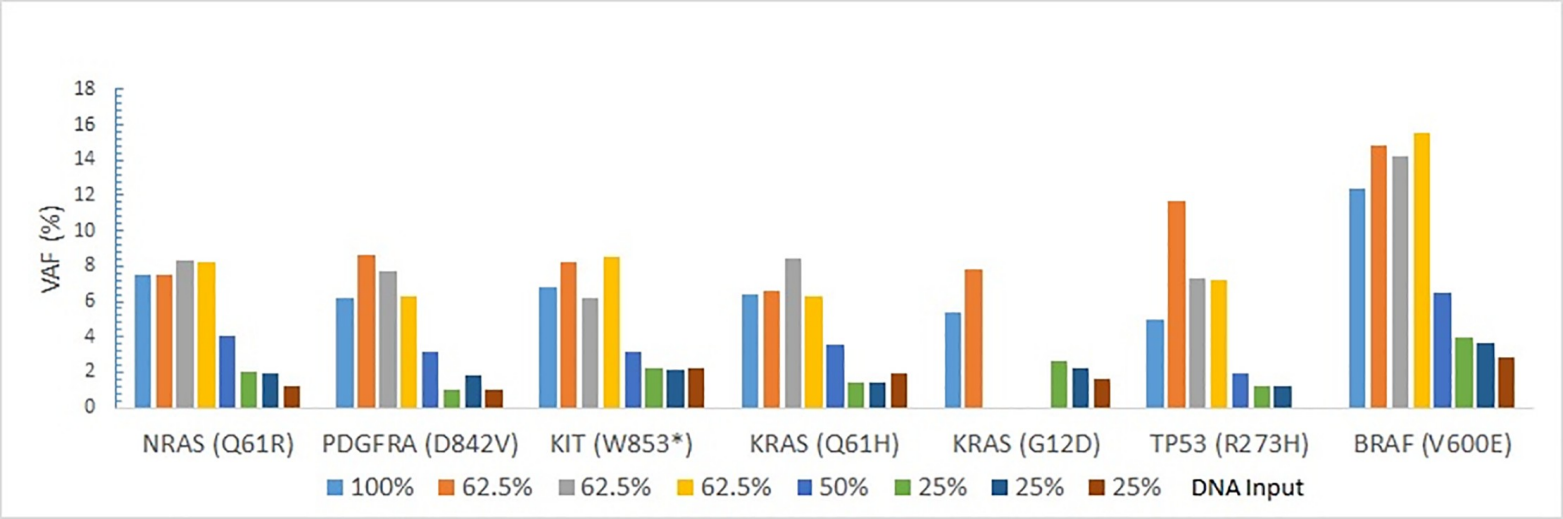
Limit of detection (LOD) study using AcroMetrix Oncology Hotspot Control sequentially diluted to 62.5%, 50%, and 25%.

The LOD study was performed using Seraseq Myeloid Mutation DNA and AcroMetrix Oncology Hotspot Control. The Seraseq Myeloid Mutation DNA consisting of 23 variants (13 were SNVs and 10 were indels/duplicaions/ITD) in 16 genes was sequenced at 100%, 50%, 25%, and 10% dilutions. The 13 SNVs were detected consistently at 100%, 50% and 25% dilutions, whereas six SNVs were detected even at 10% dilution. The *MYD88* p.L265P variant was detected till the 10% dilution with a VAF of 1.11. Of the seven indels/duplications, seven variants were detected at 100% dilution, six at 50% dilution, five at 25% dilution and 2 at 10% dilution. The *CEBPA* p.K313 V314insK was detected at 10% dilution with a VAF of 1.02 (Fig 2). The AcroMetrix Oncology Hotspot Control sample was sequenced at 100%, 62.5%, 50%, and 25%, 12.5%, 10% and 1% dilutions. The 7 SNVs were detected from 100% to 25% dilution (Fig 3).

**Fig 4:**
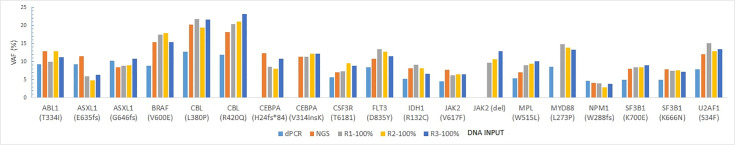
Intra-run performance using Seraseq Myeloid Mutation DNA run in triplicate.

For reproducibility studies, the intra-run reproducibility was evaluated by sequencing the Seraseq Myeloid Mutation DNA in triplicate at 100%, and the AcroMetrix Oncology Hotspot Control sample in triplicate at 25% dilution. For the Seraseq Myeloid Mutation DNA, a reproducibility of ~95% was recorded, recognizing that 2 *FLT3* ITDs and *CALR* p.L367fs*46 were not detected in the 3 replicates (Fig 4). Similarly, a high reproducibility was observed with the AcroMetrix Oncology Hotspot Control sample detecting the seven variants at a low VAF ranging from 3.93–1.01. Only a *TP53* R273H variant was not detected in the one of the triplicates at 25% dilution (the other two were detected at 1.2, 1.1 VAF) (Fig 3).

**Fig 5:**
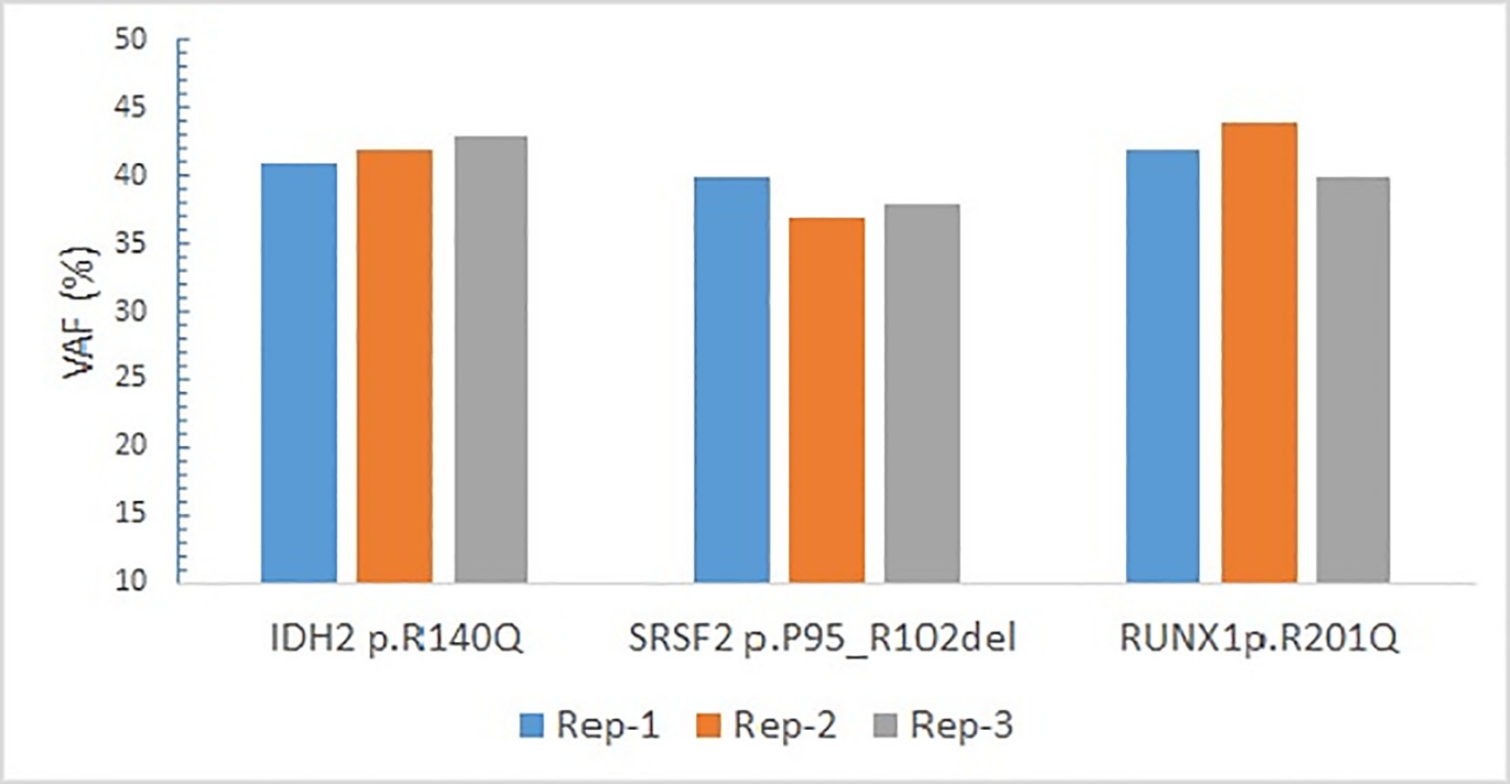
Inter-run performance using clinical samples.

The inter-run reproducibility was evaluated by sequencing a clinical sample with two SNVs and one deletion in three different runs. All the three variants were detected consistently at similar VAF and represented high reproducibility (Fig 5). Similarly, a high reproducibility was observed with the AcroMetrix Oncology Hotspot Control sample detecting the seven variants except a *KRAS* p.G12D variant not detected in two of the triplicates at 62.5% dilution (Fig 3).

### Novel variants/findings

The panel identified 880 variants across 292 genes, of which, 749 variants were in genes not covered in the 54 gene panel. The 749 variants were meticulously searched in various knowledge bases for clinical relevance. The investigation revealed 14 variants in ten genes (*PMS2*, *PDGFRB*, *PREX2*, *ATM*, *MET*, *PRKN*, *DDX41*, *KDM5C*, *KMT2C* and *HLA-A*) in patient samples that were pathogenic/likely pathogenic in AML/MDS. 96.2% (26/27) patients had at least one of these 14 novel variants. Two variants in KMT2C gene were identified to be of pathogenic significance in AML in the COSMIC database, with a Functional Analysis through Hidden Markov Models (FATHMM) score of >.90. Also, 22 variants in five genes [*ICOSLG*, *NCOR1*, *KMT2C* (10), *HLA-A* (9) and *AR*] were found to be pathogenic/ likely pathogenic in other tumor types. Overall, 13 variants were found to have a FATHMM score of >.90 in the COSMIC database (S3 Table). Further, the TMB and MSI scores ranged from 0–7 and 0–9, respectively.

## Discussion

### Interesting clinical cases

A 23-year-old male diagnosed with mixed phenotype acute leukemia demonstrated a complex cytogenetic profile. The blast count was 60%, with flow cytometry demonstrating two distinct blast populations: one population with T-cell differentiation and the other population with monocytic differentiation. The DNA sample was screened on a 54 gene myeloid NGS panel, and was reported to have *TP53* p.R248Q and *NRAS* p.G12D variants. The patient was managed with the AYA, CALGB, 10403 protocol, and had a survival of 4 months and 2 days. In the present study, the same DNA sample was used for performance evaluation of this comprehensive panel. Comprehensive analysis using the 523 gene panel identified that this patient sample had a TMB of 7 (the highest observed in this cohort). In addition, the patient’s sample was identified to have 2 novel variants in *KMT2C* gene with pathogenic significance in AML, and a FATHMM score of >.90 in COSMIC database. Additionally, it had six novel variants, five in *HLA-A* and one in *KMT2C* gene, implicated in other tumor types.

A 71-year-old male diagnosed with myeloid sarcoma had an abnormal cytogenetic profile, with loss of chromosome 7 and evidence of tetraploidy observed for *MLL*, *AML*, *ETO*, *BCR* and *ABL*. The patient’s sample was screened on a 54 gene myeloid NGS panel, and was reported to have *IDH2* p.R140Q, *JAK2* p.V617F, and *SRSF2* p.P95L variants. The patient was managed with the 7+3 regimen (7 days of cytarabine with 3 days of antracycline) and had a survival of <1 month. Comprehensive analysis using the 523 gene panel identified that this patient sample had a TMB of 3.13 and MSI of 1.69%. In addition, the patient’s sample was identified to have 4 novel variants in four genes (*HLA-A*, *KMT2C*, *PDGFRB*, and *PREX2*) with pathogenic significance in AML. The *KMT2C* variant had a FATHMM score of 1, in the COSMIC database. In addition, 5 variants in two genes (*HLA-A* and *KMT2C*) were identified, which had pathogenic significance in other tumor types.

### Panel validation

The rapid adoption of NGS based platforms into clinical diagnostic laboratories has revolutionized the field of genetic testing by creating an unprecedented opportunity to profile multiple, actionable driver genes in tumor samples [14–17]. For myeloid malignancies, the NCCN guideline recommends the patient be tested on multiplex gene panels and NGS analysis for comprehensive prognostic assessment [8]. Several reports including the TCGA study demonstrates a complex network of genetic mutations, with limited recurrent mutations [11]. The recurrent alterations in AML include genes involved in epigenetics (*TET2*, *IDH1/IDH2*, *DNMT3A*, *ASXL1*, *KMT2A*, and *EZH2*), tumor suppressor genes (*TP53*, *WT1*, and *NPM1*), oncogenes (*FLT3*, *KRAS*, *NRAS*, and *KIT*), and genes coding for transcription-differentiation (*CEBPA*, *RUNX1*) [18–20]. The list of genes altered in myeloid neoplasms is rapidly increasing, as is evident from the above mentioned profiles. Currently, the NGS panels used in clinical laboratories are limited in coverage (typically~54 genes) which leads to incomplete definition of the mutation profile, often excluding important known hot spots thus impeding identification of a complete personalized diagnosis [9, 10].

Considering the importance of comprehensive profiling, and with the decreasing cost of sequencing technologies, there has been significant interest in validating a comprehensive platform to assess clinically significant alterations in a single assay. In this pursuit, we have evaluated the clinical performance of a 523-gene comprehensive panel for screening myeloid neoplasms. The analytical performance analysis using 61 samples demonstrated the ease of use and clinical utility of TSO 500 panel. The assay had a hands-on time of ~10.5 hours and an assay time of ~3 days. The sequencing run parameters met the recommended threshold values of the manufacturer, with a high consistency among samples for each parameter (both across runs and within each run), which is evident from the low SD values represented in Fig 1. Interestingly, the run performance metric for all the nine runs in our study was found to be comparable and fully met manufacturer’s threshold recommendations. The ability to sequence both hematologic neoplasms and solid tumors simultaneously, adds substantial advantage to any clinical laboratory with respect to cost, time and efficiency.

The analytical performance of the assay demonstrated an over-all clinical sensitivity of 97.4%, specificity and, precision of 100% and accuracy of 99.9% for clinical samples. The over-all sensitivity, specificity, precision and accuracy were calculated to be 100% for Seracare Myeloid Mutation DNA, without including *FLT3* ITD and *CLAR* variants in calculations. Recent reports have highlighted that the sequencing of *CEBPA*, *CALR*, and *FLT3* genes remains a challenge on NGS platforms. The difficulty of detecting long *FLT3* ITD variants on NGS platforms has been reported previously, which might be because the chemistries employ short read sequencing (read length 50-300bp) that makes them prone to lose structural variants such as long indels [10, 21, 22]. In alignment with these reports, three *Flt3 ITD* confirmed by Sanger sequencing in three clinical samples were not detected in this current study. Also, the two *FlT3* ITD variants in the Seraseq Myeloid Mutation DNA were not detected. The same difficulty has been documented for the large deletion of 52 bp in the CALR gene [10, 22, 23]. The *CALR* 52 bp deletion was not detected by the panel in the three replicates of Seraseq Myeloid Mutation DNA. However, a variant in *CALR* gene (*CALR* c.1154_1155insTTGTC) was detected at a VAF of 36% in a clinical sample. These findings highlight the fact that the NGS panel has high coverage for these genes, but the difficulty in sequencing large indels appears to be a limitation of the sequencing chemistry. Another challenge with NGS platforms has been in sequencing GC rich genes such as *CEBPA*. The GC rich region poses a challenge in amplification during library preparation for sequencing [23]. However, we were able to detect *CEBPA* variants in both clinical as well as Seraseq Myeloid Mutation DNA samples. In the Seraseq Myeloid Mutation DNA sample, two *CEBPA* variant were detected upto the 25% dilution and one variant was detected even at a dilution of 10% with a VAF of 1.02.

The LOD studies with Seraseq Myeloid Mutation DNA and AcroMetrix Oncology Hotspot Control samples demonstrated that both SNVs and indels could be detected at low VAF of ~1%.

The SNVs were detected consistently at low VAF ~2%, as observed with the detection of all 7 variants at 25% dilution (in triplicate) of the AcroMetrix Oncology Hotspot Control. High intra-run reproducibility was demonstrated with Seraseq Myeloid Mutation DNA and AcroMetrix Oncology Hotspot Control sequenced in triplicate at 100% and 25% dilutions, respectively. High inter-run reproducibility was demonstrated with clinical sample and AcroMetrix Oncology Hotspot Control sequenced in triplicate at 100% and 62.5% dilutions, respectively.

In addition to the evaluation of the analytical performance of the platform, the clinical utility of comprehensive analysis in myeloid neoplasms was assessed. The molecular heterogeneity of the tumor was evident from the 3756 variants (880 unique variants) identified across 292 genes in the clinical samples. Similar findings were observed in the WES based study by TCGA Research Network on 200 AML patients, which identified variants across 237 genes [11]. Interestingly, of the 880 variants identified in this assay, only 14.8% (131) of these variants were in genes that are covered in the 54 gene panel. The remaining 85.2% (749) of variants were observed in genes that are not covered in myeloid panels routinely employed in clinical diagnostic laboratories. On investigating these 749 variants, 14 variants in ten genes were found to be pathogenic/ likely pathogenic in myeloid neoplasms and at least one variant was present in 96.2% (26/27) patients included in this study. Further, 22 variants in five genes were found to be pathogenic/ likely pathogenic in other tumor types and were present in 96.2% of patients included in this study. Another method employed to evaluate the pathogenicity of these variants was to document the FATHMM score. The scores are based on the algorithm that predicts the functional, molecular and phenotypic consequences of protein missense variants using Hidden Markov models. The functional scores for individual mutations from FATHMM-MKL are in the form of a single p-value, ranging from 0 to 1. Scores above 0.5 are deleterious, but in order to highlight the most significant data in COSMIC, only scores ≥ 0.7 are classified as 'pathogenic'. Interestingly, 13 variants were found to have a FATHMM score of >.90, in the COSMIC database.

In addition to the exhaustive alterations observed with comprehensive profiling, TMB and MSI were also recorded. TMB is an emerging biomarker of sensitivity to immune checkpoint inhibitors (ICI). Chalmers et al. has documented TMB across a diverse cohort of 100,000 cancer cases and tested for association between somatic alterations and TMB in over 100 tumor types [12]. Of these 100 tumor types, myeloid neoplasms had the least average TMB compared to other tumor types. In 888 AML cases, an average TMB of 1.7 was observed, with the highest TMB of 15 [12]. In our cohort, an average TMB of 2.0 was observed with the highest of 7.

The novel variants and TMB observed in the study were not statistically significant due to the small sample size, however interesting correlations with clinical parameters were observed. The case with a survival of four months and two days was identified to have a TMB of 7 (the highest observed in this cohort). In addition, the case had two novel variants that correlated with the high TMB, as reported by Chalmers et al. Further, the patient’s sample had several novel variants with pathogenic significance in myeloid neoplasms and other tumor types. Another case which had a survival of <1 month had similar observations with several novel variants of pathogenic significance in myeloid neoplasms and other tumor types.

Unlike solid tumors where TMB and/or programmed death ligand 1 (PD-L1) expression have been validated as biomarkers to identify patients who may benefit from ICI, their utility in AML and MDS is yet to be established [24]. Nevertheless, several studies have reported benefit of ICI in combination with standard therapies including hypomethylating agents in AML and MDS, though the mechanism appears to be enhancement of graft-versus-tumor effect and is independent of TMB [25, 26]. Cut-off values for TMB vary considerably across histological types of malignancies. Generally, higher overall response rate and durable response rates, are seen in tumors with high TMB. Exceptions to the rule such as Merkel-cell carcinomas that exhibit low TMB and yet show response to ICI have been identified [27]. Therefore, although myeloid neoplasms typically show low TMB, the predictive and/or prognostic potential for this biomarker requires further study perhaps with lower TMB thresholds. As regards to MSI, our study results are in agreement with previously published data that MSI is rare in AML [28].

Approximately 15–40% of patients do not achieve complete remission after induction and most AML patients will have a relapse within 3 years after diagnosis [19]. Second line gene panels for AML, that have a wider coverage but short of whole exome sequencing may prove useful for the management of AML. Such a comprehensive panel may be a particularly important tool for investigating these group of patients for identifying additional biomarkers that may not be identifiable using smaller panels.

In conclusion, the comprehensive panel employed in this study, demonstrates its ease of use and clinical utility for myeloid neoplasms. The panel has extensive coverage across the entire genome, for variants significantly beyond those captured on existing NGS platforms for hematological malignancies. However, the platform still faces challenge with sequencing long Indels (*CALR*, and *FLT3* ITD), which seem to be a limitation of sequencing chemistry. Importantly, in assessing the clinical utility, the assay revealed novel variants that might have diagnostic, prognostic, and/or therapeutic significance in myeloid neoplasms. This study was limited by the fact that the panel used in this study did not contain RNA sequencing data and therefore the performance for translocation analysis is yet to be determined. In addition, secondary analysis and reporting pipeline used may have been insufficient for analysis. With inclusion of RNA sequencing and optimization of pipelines for analysis, this comprehensive assay shows potential in revealing new prognostic markers and, with further research, potential therapeutic targets that may facilitate personalized therapy.

## Supporting information

S1 Table Detailed QC Metric across 4 runs performed to evaluate the clinical performance of TSO-500. (DOCX)

S2 Table The list of variants detected by the platform used for calculating performance metric. (DOCX)

S3 Table List of novel variants identified with pathogenic significance in myeloid neoplasms or other tumor types with correlation to clinical parameters. (DOCX)
