# Supplementary material for: Clinical performance and utility of a comprehensive next-generation sequencing DNA panel for the simultaneous analysis of variants, TMB and MSI for myeloid neoplasms
Source: PLoS One. 2020 Oct 19;15(10):e0240976. doi: 10.1371/journal.pone.0240976 (PMC7571681; doi:10.1371/journal.pone.0240976)
Supplement: S1 Table — (DOCX) [file pone.0240976.s001.docx]

S1 Table. Detailed QC Metric across 4 runs performed to evaluate the clinical performance of TSO-500.

| [Run Metrics] | | | | |
| --- | --- | --- | --- | --- |
| Runs | Run 1 | Run 2 | Run 3 | Run 4 |
| PCT_PF_READS (%) | 88.6 | 90.6 | 89.5 | 91.8 |
| PCT_Q30_R1 (%) | 93.1 | 93.9 | 93.2 | 94.5 |
| PCT_Q30_R2 (%) | 90.6 | 91.7 | 90.7 | 92.2 |
| [DNA Library QC Metrics for Small Variant Calling and TMB] | | | | |
| MEDIAN_INSERT_SIZE (bp) | 123.6 | 127.3 | 126.8 | 123.5 |
| MEDIAN_EXON_COVERAGE (Count) | 1051.2 | 1296.8 | 1237.9 | 1244.7 |
| PCT_EXON_50X (%) | 99.32 | 99.3 | 99.3 | 99.5 |
|  |  |  |  |  |
| [DNA Library QC Metrics for MSI] | | | | |
| USABLE_MSI_SITES (Count) | 120.6 | 120.8 | 119.9 | 116.1 |
| TOTAL_PF_READS (Count) | 76189720.4 | 82225779.8 | 79096773.8 | 82729863.6 |
| MEAN_FAMILY_SIZE (Count) | 1.6 | 1.4 | 1.4 | 1.4 |
| MEDIAN_TARGET_COVERAGE (Count) | 1005.6 | 1240.9 | 1179.4 | 1189.8 |
| PCT_CHIMERIC_READS (%) | 0.13 | 0.1 | 0.1 | 0.1 |
| PCT_EXON_100X (%) | 98.91 | 98.9 | 99.0 | 99.2 |
| PCT_READ_ENRICHMENT (%) | 83.84 | 82.0 | 82.7 | 82.7 |
| PCT_USABLE_UMI_READS (%) | 99.7 | 99.7 | 99.7 | 99.7 |
| MEAN_TARGET_COVERAGE (Count) | 991.82 | 1238.0 | 1174.4 | 1180.8 |
| PCT_ALIGNED_READS (%) | 97.58 | 98.0 | 97.4 | 96.9 |
| PCT_CONTAMINATION_EST (%) | 0.06 | 0.0 | 0.0 | 0.0 |
| PCT_PF_UQ_READS (%) | 100 | 100.0 | 100.0 | 100.0 |
| PCT_TARGET_0.4X_MEAN (%) | 92.3 | 91.9 | 91.9 | 92.2 |
| PCT_TARGET_100X (%) | 97.96 | 98.1 | 98.0 | 98.1 |
| PCT_TARGET_250X (%) | 95.39 | 96.2 | 96.1 | 96.3 |

Table S2. The list of variants detected by the platform used for calculating performance metric

| **Seraseq Myeloid Mutation DNA** | **AcroMetrix Oncology Hotspot Control** | **Myeloid neoplasm control samples** | **Clinical samples** | | |
| --- | --- | --- | --- | --- | --- |
|  |  |  | *ASXL1* p.E635Rfs*15 | *KRAS* p.G12V | *TET2* p.Y1679* |
| *ABL1* (T334I) | *NRAS* p.Q61R | BRAF (V600E) | *ASXL1* p.Y591 | *KRAS* p.G13D | *TP53* c.559+1G>A |
| *ASXL1* (E635fs) | *PDGFRA* p.D842V | DNMT3A p.R882C | *CALR* p.K385Nfs | *KRAS* p.K117N | *TP53*  p.R248Q |
| *ASXL1* (G646fs) | *KIT* p.W853* | DNMT3A p.R882H | *CBL* p.C358Y p.C404Y | *NRAS* p.G12D | *U2AF1* p.S34F; |
| *BRAF* (V600E) | *KRAS* p.Q61H | DNMT3A p.L547R | *CEBPA*: p.P189_P189del | *NOTCH1* p.P2137S | *U2AF1* Q157R |
| *CBL* (L380P) | *KRAS* p.G12D | FLT3 p.D835N | *DNMT3A* p.G327Rp.G361R p.G550R | *NPM1* p.W288Cfs | *ZRSR2* p.S447_R448dup |
| *CBL* (R420Q) | *TP53* p.R273H | JAK2 p.V617F | *DNMT3A* p.R882C | *NRAS* p.G12D |  |
| *CEBPA* (H24fs*84) | *BRAF* p.V600E | KIT (W853*) | *DNMT3A* p.R882H | *NRAS* p.G12S. |  |
| *CEBPA* (V314insK) |  | KRAS (G12D) | *DNMT3A*: p.L547R | *NRAS* p.G13R |  |
| *CSF3R* (T6181) |  | KRAS (Q61H) | *EZH2* p.N268Mfs* | *PHF6* p.I280Tp.I314Tp.I315T |  |
| *FLT3* (D835Y) |  | NRAS (Q61R) | *FLT3* 13q12.2 ITD | *PTPN11* p.A72T |  |
| *IDH1* (R132C) |  | NRAS p.G12D | *FLT3* p.D600_L601insRGREYEYD | *RUNX1* p.L29S p.L44S p.L56S " |  |
| *JAK2* (V617F) |  | PDGFRA (D842V) | *FLT3* p.D835H | *RUNX1* p.R166* |  |
| *JAK2* (c.1624_1629delAATGAA) |  | TET2 p.Q770* | *FLT3* p.D835N | *RUNX1* p.R201Q |  |
| *MPL* (W515L) |  | TET2 p.R550* | *FLT3* p.D835V | *SF3B1* p.H516Q p.H662Q. |  |
| *MYD88* (L273P) |  | TP53 p.A159P | *FLT3* p.D835Y | *SRSF2* p.P95_R102del |  |
| *NPM1* (W288fs) |  | TP53 p.R248Q | FLT3 p.V592_Y599dup | SRSF2 p.P96L |  |
| *SF3B1* (K700E) |  | TP53 (R273H) | GATA2 p.P161A | SRSF2 p.P95L |  |
| *SRSF2* (p.P95 R102del) |  |  | IDH1 p.R132H | TET2 p.Q770* |  |
| *SF3B1* (K666N) |  |  | IDH2 p.R140Q | TET2 p.Q810R |  |
| *U2AF1* (S34F) |  |  | JAK2 p.V617F | TET2 p.R550* |  |

Table S3. List of novel variants identified with pathogenic significance in myeloid neoplasms or other tumor types with correlation to clinical parameters.

|  | | | | | Sex | | Age | | Cytogenetics | | Management | |
| --- | --- | --- | --- | --- | --- | --- | --- | --- | --- | --- | --- | --- |
| Coordinates | Gene | N | Pathogenic in following tumor type(s) | FATHMM Score | M | F | <68 | >68 | N | A | T | NT |
| c.170T>A | AR | 8 | Lung, Large Intestine, Thyroid, Liver, Prostate | 0.03 | 6 | 2 | 5 | 3 | 6 | 2 | 2 | 6 |
| c.228A>G | HLA-A | 20 | Upper aerodigestive tract (UAT), Prostrate, Soft Tissue, CNS, Hematopoietic and Lymphoid | 0 | 13 | 7 | 12 | 8 | 10 | 10 | 6 | 14 |
| c.605C>T | HLA-A | 19 | Soft tissue, UST, CNS, urinary tract and thyroid) | 0.03 | 14 | 5 | 11 | 8 | 9 | 10 | 6 | 13 |
| c.233A>G | HLA-A | 18 | Prostate, CNS, UAT, Lung, Breast | 0.01 | 12 | 6 | 11 | 7 | 9 | 9 | 5 | 13 |
| c.506G>A | HLA-A | 14 | UAT, Soft Tissue, CNS, pancreas, Thyroid) | 0.07 | 10 | 4 | 8 | 6 | 6 | 8 | 5 | 9 |
| c.808G>T | HLA-A | 11 | Soft Tissue, CNS, UAT, Lung | 0.09 | 5 | 5 | 7 | 3 | 6 | 4 | 6 | 4 |
| c.142G>T | HLA-A | 10 | Thyroid, CNS, UAT, Skin, Lung | 0 | 6 | 4 | 7 | 3 | 6 | 4 | 3 | 7 |
| c.691G>A | HLA-A | 7 | UAT, Soft Tissue, Large Intestine, Skin, Hematopoietic and Lymphoid) | - | 5 | 2 | 5 | 2 | 5 | 2 | 2 | 5 |
| c.10A>G | HLA-A | 3 | UAT, Soft Tissue, Large Intestine, Skin, Hematopoietic and Lymphoid) | 0 | 2 | 1 | 2 | 1 | 1 | 2 | 2 | 1 |
| c.239G>A | HLA-A | 3 | UAT, Soft Tissue, Lung, Skin, Thyroid) | 0 | 2 | 1 | 2 | 1 | 1 | 2 | 2 | 1 |
| c.934A>C | HLA-A | 3 | UAT, CNS , Soft Tissue, Breast, Skin | 0.02 | 2 | 1 | 2 | 1 | 1 | 2 | 2 | 1 |
| c.806C>T | HLA-A | 2 | Soft Tissue, Large Intestine, CNS, Thyroid | 0.12 | 1 | 1 | 1 | 1 | 1 | 1 | 1 | 1 |
| c.497T>C | HLA-A | 1 | UAT, CNS, thyroid | 0.18 | 0 | 1 | 0 | 1 | 1 | 0 | 1 | 0 |
| c.898+427T>C | ICOSLG | 26 | Thyroid, Biliary Tract, Prostrate, Large Intestine and Breast | 0.01 | 16 | 9 | 14 | 11 | 13 | 12 | 8 | 17 |
| c.2959T>C | KMT2C | 26 | Soft tissue, UST, CNS, Urinary Tract and Thyroid) | 0.99 | 17 | 8 | 13 | 12 | 12 | 13 | 7 | 18 |
| c.2656C>T | KMT2C | 23 | Thyroid, Oesophagus, Bone (Osteosarcoma) and Large Intestine | 0.99 | 14 | 8 | 12 | 10 | 11 | 11 | 8 | 14 |
| c.1173C>A | KMT2C | 17 | Soft Tissue, Hematopoietic and Lymphoid, CNS, UAT, Liver | 0.97 | 9 | 7 | 8 | 8 | 9 | 7 | 5 | 11 |
| c.925C>T | KMT2C | 13 | Soft Tissue, Large Intestine, CNS, UAT, Thyroid | 0.99 | 8 | 5 | 8 | 5 | 5 | 8 | 4 | 9 |
| c.1042G>A | KMT2C | 12 | Soft Tissue, Large Intestine, Thyroid, Pancreas, Hematopoietic and Lymphoid | 1 | 10 | 2 | 7 | 5 | 6 | 6 | 3 | 9 |
| c.2681G>A | KMT2C | 6 | Soft Tissue, Thyroid, Skin, Breast, UAT | 0.99 | 5 | 1 | 5 | 1 | 3 | 3 | 2 | 4 |
| c.2968A>G | KMT2C | 5 | Soft Tissue, CNS, Skin, Pancreas, Hematopoietic and Lymphoid | 0.9 | 4 | 1 | 3 | 2 | 3 | 2 | 2 | 3 |
| c.2578C>T | KMT2C | 4 | Thyroid, Large Intestine, Lung | 0.96 | 3 | 1 | 1 | 3 | 1 | 3 | 1 | 3 |
| c.943G>A | KMT2C | 4 | Soft Tissue, CNS, Liver, Lung, Prostate | 0.98 | 3 | 1 | 2 | 2 | 1 | 3 | 1 | 3 |
| c.2459C>T | KMT2C | 2 | Large Intestine, Urinary Tract, Liver, Hematopoietic and Lymphoid, Bone | 0.99 | 0 | 2 | 0 | 2 | 1 | 1 | 0 | 2 |
| c.2573G>T | KMT2C | 1 | Thyroid, Large Intestine, Lung | 0.98 | 1 | 0 | 0 | 1 | 0 | 1 | 0 | 1 |
| c.5053G>T | KMT2C | 1 | Thyroid, Large Intestine, CNS, Prostate, Kidney | 0.99 | 1 | 0 | 1 | 0 | 1 | 0 | 0 | 1 |
| c.59A>C | NCOR1 | 22 | UAT, Breast, CNS, Thyroid, Pancreas) | 0.91 | 15 | 6 | 12 | 9 | 10 | 11 | 4 | 17 |
| c.2186_2187delTC | PMS2 | 1 | Myeloid neoplasms | - | 1 | 0 | 0 | 1 | 0 | 1 | 0 | 1 |
| c.3287C>T | PDGFRB | 1 | Myeloid neoplasms | - | 1 | 0 | 0 | 1 | 0 | 1 | 0 | 1 |
| c.2340_2341delAG | PREX2 | 1 | Myeloid neoplasms | - | 1 | 0 | 0 | 1 | 0 | 1 | 0 | 1 |
| c.1229T>C | ATM | 1 | Myeloid neoplasms | - | 1 | 0 | 1 | 0 | 1 | 0 | 0 | 1 |
| c.2962C>T | MET | 2 | Myeloid neoplasms | - | 1 | 1 | 1 | 1 | 1 | 1 | 1 | 1 |
| c.823C>T | PRKN | 1 | Myeloid neoplasms | - | 1 | 0 | 0 | 1 | 1 | 0 | 0 | 1 |
| c.415_418dupGATG | DDX41 | 1 | Myeloid neoplasms | - | 1 | 0 | 0 | 1 | 1 | 0 | 1 | 0 |
| c.1574G>A | DDX41 | 1 | Myeloid neoplasms | - | 1 | 0 | 0 | 1 | 1 | 0 | 1 | 0 |
|  | KDM5C | 1 | Myeloid neoplasms | - | 1 | 0 | 1 | 0 | 1 | 0 | 0 | 1 |
