# Supplementary material for: Clinical performance and utility of a comprehensive next-generation sequencing DNA panel for the simultaneous analysis of variants, TMB and MSI for myeloid neoplasms
Source: PLoS One. 2020 Oct 19;15(10):e0240976. doi: 10.1371/journal.pone.0240976 (PMC7571681; doi:10.1371/journal.pone.0240976)
Supplement: S2 Table — (DOCX) [file pone.0240976.s002.docx]

S2 Table. The list of variants detected by the platform used for calculating performance metric

| **Seraseq Myeloid Mutation DNA** | **AcroMetrix Oncology Hotspot Control** | **Myeloid neoplasm control samples** | **Clinical samples** | | |
| --- | --- | --- | --- | --- | --- |
|  |  |  | *ASXL1* p.E635Rfs*15 | *KRAS* p.G12V | *TET2* p.Y1679* |
| *ABL1* (T334I) | *NRAS* p.Q61R | BRAF (V600E) | *ASXL1* p.Y591 | *KRAS* p.G13D | *TP53* c.559+1G>A |
| *ASXL1* (E635fs) | *PDGFRA* p.D842V | DNMT3A p.R882C | *CALR* p.K385Nfs | *KRAS* p.K117N | *TP53*  p.R248Q |
| *ASXL1* (G646fs) | *KIT* p.W853* | DNMT3A p.R882H | *CBL* p.C358Y p.C404Y | *NRAS* p.G12D | *U2AF1* p.S34F; |
| *BRAF* (V600E) | *KRAS* p.Q61H | DNMT3A p.L547R | *CEBPA*: p.P189_P189del | *NOTCH1* p.P2137S | *U2AF1* Q157R |
| *CBL* (L380P) | *KRAS* p.G12D | FLT3 p.D835N | *DNMT3A* p.G327Rp.G361R p.G550R | *NPM1* p.W288Cfs | *ZRSR2* p.S447_R448dup |
| *CBL* (R420Q) | *TP53* p.R273H | JAK2 p.V617F | *DNMT3A* p.R882C | *NRAS* p.G12D |  |
| *CEBPA* (H24fs*84) | *BRAF* p.V600E | KIT (W853*) | *DNMT3A* p.R882H | *NRAS* p.G12S. |  |
| *CEBPA* (V314insK) |  | KRAS (G12D) | *DNMT3A*: p.L547R | *NRAS* p.G13R |  |
| *CSF3R* (T6181) |  | KRAS (Q61H) | *EZH2* p.N268Mfs* | *PHF6* p.I280Tp.I314Tp.I315T |  |
| *FLT3* (D835Y) |  | NRAS (Q61R) | *FLT3* 13q12.2 ITD | *PTPN11* p.A72T |  |
| *IDH1* (R132C) |  | NRAS p.G12D | *FLT3* p.D600_L601insRGREYEYD | *RUNX1* p.L29S p.L44S p.L56S " |  |
| *JAK2* (V617F) |  | PDGFRA (D842V) | *FLT3* p.D835H | *RUNX1* p.R166* |  |
| *JAK2* (c.1624_1629delAATGAA) |  | TET2 p.Q770* | *FLT3* p.D835N | *RUNX1* p.R201Q |  |
| *MPL* (W515L) |  | TET2 p.R550* | *FLT3* p.D835V | *SF3B1* p.H516Q p.H662Q. |  |
| *MYD88* (L273P) |  | TP53 p.A159P | *FLT3* p.D835Y | *SRSF2* p.P95_R102del |  |
| *NPM1* (W288fs) |  | TP53 p.R248Q | FLT3 p.V592_Y599dup | SRSF2 p.P96L |  |
| *SF3B1* (K700E) |  | TP53 (R273H) | GATA2 p.P161A | SRSF2 p.P95L |  |
| *SRSF2* (p.P95 R102del) |  |  | IDH1 p.R132H | TET2 p.Q770* |  |
| *SF3B1* (K666N) |  |  | IDH2 p.R140Q | TET2 p.Q810R |  |
| *U2AF1* (S34F) |  |  | JAK2 p.V617F | TET2 p.R550* |  |
