# Supplementary material for: Clinical performance and utility of a comprehensive next-generation sequencing DNA panel for the simultaneous analysis of variants, TMB and MSI for myeloid neoplasms
Source: PLoS One. 2020 Oct 19;15(10):e0240976. doi: 10.1371/journal.pone.0240976 (PMC7571681; doi:10.1371/journal.pone.0240976)
Supplement: S3 Table — (DOCX) [file pone.0240976.s003.docx]

S3 Table. List of novel variants identified with pathogenic significance in myeloid neoplasms or other tumor types with correlation to clinical parameters.

|  | | | | | Sex | | Age | | Cytogenetics | | Management | |
| --- | --- | --- | --- | --- | --- | --- | --- | --- | --- | --- | --- | --- |
| Coordinates | Gene | N | Pathogenic in following tumor type(s) | FATHMM Score | M | F | <68 | >68 | N | A | T | NT |
| c.170T>A | AR | 8 | Lung, Large Intestine, Thyroid, Liver, Prostate | 0.03 | 6 | 2 | 5 | 3 | 6 | 2 | 2 | 6 |
| c.228A>G | HLA-A | 20 | Upper aerodigestive tract (UAT), Prostrate, Soft Tissue, CNS, Hematopoietic and Lymphoid | 0 | 13 | 7 | 12 | 8 | 10 | 10 | 6 | 14 |
| c.605C>T | HLA-A | 19 | Soft tissue, UST, CNS, urinary tract and thyroid) | 0.03 | 14 | 5 | 11 | 8 | 9 | 10 | 6 | 13 |
| c.233A>G | HLA-A | 18 | Prostate, CNS, UAT, Lung, Breast | 0.01 | 12 | 6 | 11 | 7 | 9 | 9 | 5 | 13 |
| c.506G>A | HLA-A | 14 | UAT, Soft Tissue, CNS, pancreas, Thyroid) | 0.07 | 10 | 4 | 8 | 6 | 6 | 8 | 5 | 9 |
| c.808G>T | HLA-A | 11 | Soft Tissue, CNS, UAT, Lung | 0.09 | 5 | 5 | 7 | 3 | 6 | 4 | 6 | 4 |
| c.142G>T | HLA-A | 10 | Thyroid, CNS, UAT, Skin, Lung | 0 | 6 | 4 | 7 | 3 | 6 | 4 | 3 | 7 |
| c.691G>A | HLA-A | 7 | UAT, Soft Tissue, Large Intestine, Skin, Hematopoietic and Lymphoid) | - | 5 | 2 | 5 | 2 | 5 | 2 | 2 | 5 |
| c.10A>G | HLA-A | 3 | UAT, Soft Tissue, Large Intestine, Skin, Hematopoietic and Lymphoid) | 0 | 2 | 1 | 2 | 1 | 1 | 2 | 2 | 1 |
| c.239G>A | HLA-A | 3 | UAT, Soft Tissue, Lung, Skin, Thyroid) | 0 | 2 | 1 | 2 | 1 | 1 | 2 | 2 | 1 |
| c.934A>C | HLA-A | 3 | UAT, CNS , Soft Tissue, Breast, Skin | 0.02 | 2 | 1 | 2 | 1 | 1 | 2 | 2 | 1 |
| c.806C>T | HLA-A | 2 | Soft Tissue, Large Intestine, CNS, Thyroid | 0.12 | 1 | 1 | 1 | 1 | 1 | 1 | 1 | 1 |
| c.497T>C | HLA-A | 1 | UAT, CNS, thyroid | 0.18 | 0 | 1 | 0 | 1 | 1 | 0 | 1 | 0 |
| c.898+427T>C | ICOSLG | 26 | Thyroid, Biliary Tract, Prostrate, Large Intestine and Breast | 0.01 | 16 | 9 | 14 | 11 | 13 | 12 | 8 | 17 |
| c.2959T>C | KMT2C | 26 | Soft tissue, UST, CNS, Urinary Tract and Thyroid) | 0.99 | 17 | 8 | 13 | 12 | 12 | 13 | 7 | 18 |
| c.2656C>T | KMT2C | 23 | Thyroid, Oesophagus, Bone (Osteosarcoma) and Large Intestine | 0.99 | 14 | 8 | 12 | 10 | 11 | 11 | 8 | 14 |
| c.1173C>A | KMT2C | 17 | Soft Tissue, Hematopoietic and Lymphoid, CNS, UAT, Liver | 0.97 | 9 | 7 | 8 | 8 | 9 | 7 | 5 | 11 |
| c.925C>T | KMT2C | 13 | Soft Tissue, Large Intestine, CNS, UAT, Thyroid | 0.99 | 8 | 5 | 8 | 5 | 5 | 8 | 4 | 9 |
| c.1042G>A | KMT2C | 12 | Soft Tissue, Large Intestine, Thyroid, Pancreas, Hematopoietic and Lymphoid | 1 | 10 | 2 | 7 | 5 | 6 | 6 | 3 | 9 |
| c.2681G>A | KMT2C | 6 | Soft Tissue, Thyroid, Skin, Breast, UAT | 0.99 | 5 | 1 | 5 | 1 | 3 | 3 | 2 | 4 |
| c.2968A>G | KMT2C | 5 | Soft Tissue, CNS, Skin, Pancreas, Hematopoietic and Lymphoid | 0.9 | 4 | 1 | 3 | 2 | 3 | 2 | 2 | 3 |
| c.2578C>T | KMT2C | 4 | Thyroid, Large Intestine, Lung | 0.96 | 3 | 1 | 1 | 3 | 1 | 3 | 1 | 3 |
| c.943G>A | KMT2C | 4 | Soft Tissue, CNS, Liver, Lung, Prostate | 0.98 | 3 | 1 | 2 | 2 | 1 | 3 | 1 | 3 |
| c.2459C>T | KMT2C | 2 | Large Intestine, Urinary Tract, Liver, Hematopoietic and Lymphoid, Bone | 0.99 | 0 | 2 | 0 | 2 | 1 | 1 | 0 | 2 |
| c.2573G>T | KMT2C | 1 | Thyroid, Large Intestine, Lung | 0.98 | 1 | 0 | 0 | 1 | 0 | 1 | 0 | 1 |
| c.5053G>T | KMT2C | 1 | Thyroid, Large Intestine, CNS, Prostate, Kidney | 0.99 | 1 | 0 | 1 | 0 | 1 | 0 | 0 | 1 |
| c.59A>C | NCOR1 | 22 | UAT, Breast, CNS, Thyroid, Pancreas) | 0.91 | 15 | 6 | 12 | 9 | 10 | 11 | 4 | 17 |
| c.2186_2187delTC | PMS2 | 1 | Myeloid neoplasms | - | 1 | 0 | 0 | 1 | 0 | 1 | 0 | 1 |
| c.3287C>T | PDGFRB | 1 | Myeloid neoplasms | - | 1 | 0 | 0 | 1 | 0 | 1 | 0 | 1 |
| c.2340_2341delAG | PREX2 | 1 | Myeloid neoplasms | - | 1 | 0 | 0 | 1 | 0 | 1 | 0 | 1 |
| c.1229T>C | ATM | 1 | Myeloid neoplasms | - | 1 | 0 | 1 | 0 | 1 | 0 | 0 | 1 |
| c.2962C>T | MET | 2 | Myeloid neoplasms | - | 1 | 1 | 1 | 1 | 1 | 1 | 1 | 1 |
| c.823C>T | PRKN | 1 | Myeloid neoplasms | - | 1 | 0 | 0 | 1 | 1 | 0 | 0 | 1 |
| c.415_418dupGATG | DDX41 | 1 | Myeloid neoplasms | - | 1 | 0 | 0 | 1 | 1 | 0 | 1 | 0 |
| c.1574G>A | DDX41 | 1 | Myeloid neoplasms | - | 1 | 0 | 0 | 1 | 1 | 0 | 1 | 0 |
|  | KDM5C | 1 | Myeloid neoplasms | - | 1 | 0 | 1 | 0 | 1 | 0 | 0 | 1 |
